# Trends in patient tobacco use behaviors as reported by tobacco treatment providers: Findings from a repeated crosssectional survey

**DOI:** 10.18332/tpc/210929

**Published:** 2025-10-20

**Authors:** Melissa Mercincavage, Patrick V. Barnwell, Michelle Kennedy, Ollie Ganz, Cristine D. Delnevo, Michael B. Steinberg

**Affiliations:** 1 Division of General Internal Medicine, Department of Medicine, Rutgers Robert Wood Johnson Medical School, New Brunswick, United States; 2 Rutgers Institute for Nicotine and Tobacco Studies, New Brunswick, United States; 3 Rutgers School of Public Health, New Brunswick, United States

**Keywords:** tobacco use, tobacco surveillance, cessation, tobacco treatment providers, smoking

**Dear Editor,**

The tobacco marketplace is more varied than ever, giving consumers a breadth of products to choose from, including cigarettes, cigars, smokeless tobacco, electronic cigarettes, and nicotine pouches^1^. Product diversity has resulted in increasingly complex patterns of tobacco use, including experimentation with new products and multiple tobacco product use^2,3^, and a more complicated policy landscape where regulations differ by product^4,5^. Tobacco treatment providers (i.e. individuals providing tobacco treatment services) may be among the first to notice changes in patients’ quitting or shifts in tobacco product use behaviors resulting from the availability of new products or policy changes^6^. Accordingly, the Rutgers Center for Rapid Surveillance of Tobacco (CRST) conducts bi-annual cross-sectional surveys of the Association for the Treatment of Tobacco Use and Dependence (ATTUD), a large professional organization of primarily US-based tobacco treatment providers, for signal generation of such changes among their clients. Here, we report observations about provider-reported patient tobacco use behaviors collected during the first two survey waves.

We solicited participation from ATTUD members via email requests through the organization’s *listserv* to complete a short, online survey hosted on the Qualtrics platform during January–March 2024 (Wave 1) and November– December 2024 (Wave 2) (Supplementary file). Eligible participants indicated currently providing tobacco and/or nicotine product treatment to patients in the US and consented to participate in the survey. Wave 1 participants received no compensation; to increase participation in Wave 2, all those who completed the survey were offered a $25 Amazon gift card.

Fifty-one and ninety-two tobacco treatment providers completed Wave 1 and Wave 2, respectively. They were geographically diverse, 60% had graduate degrees, and 77% were Certified Tobacco Treatment Specialists. Table 1 illustrates, across both waves, the percentage of tobacco treatment providers reporting in the past 6 months if patients: 1) mentioned a particular tobacco product, 2) reported seeking cessation from a particular tobacco product, and 3) reported using a particular tobacco product to support quitting another product. Of note, across both waves, combustible and electronic cigarettes were the most common products that providers reported their patients sought to quit using (>90%), followed by smokeless tobacco (>70%), and cigars (>50%). E-cigarettes were also the most commonly reported product that providers observed patients using to quit another tobacco product (>90%), followed by nicotine pouches (>50%). In response to open-ended questions asking providers to share about their clinical encounters, several providers expressed concerns about the nicotine concentrations and lack of regulation of vaping products, as well as the increased availability and use of nicotine pouches.

**Table 1:** Past 6-month patient tobacco and/or nicotine product use behaviors (%), as reported by U.S. tobacco treatment providers

*Products*	*Patients mentioned product*		*Patients sought treatment to stop using THIS product*		*Patients used this product to stop using ANOTHER product*	
	*Wave 1*	*Wave 2*	*Wave 1*	*Wave 2*	*Wave 1*	*Wave 2*
Cigarettes	**98.1**	**94.5**	**94.1**	**95.6**	11.5	25.8
E-cigarettes	**96.2**	**97.8**	**90.2**	**92.3**	**92.3**	**92.2**
Cigars	73.1	76.9	51.0	79.1	21.2	25.6
Smokeless tobacco	73.1	81.3	70.6	75.8	34.6	23.3
Nicotine pouches	75.0	75.8	27.5	36.3	**57.7**	**56.7**
Heated tobacco products	34.6	33.0	23.5	16.7	17.3	12.4

Data are given as percentages (%). Bolded typeface indicates products with the greatest endorsement by providers. Data were collected during January–March 2024 (Wave 1, n=51) and November–December 2024 (Wave 2, n=92).

In summary, surveillance of tobacco treatment providers indicates high rates of treatment seeking for e-cigarettes, smokeless, and cigars, as well as high rates of e-cigarette and oral nicotine product use to quit other tobacco products. These patterns have been noted in population data^7,8^. People are increasingly seeking treatment for e-cigarette dependence and using nicotine pouches as smoking cessation aids. As evidence grows supporting the potential cessation benefits of other tobacco products^1,9,10^, continued surveillance of providers as sentinels may assist in informing regulatory efforts.

## Supplementary Material



## Data Availability

The data supporting this research are available from the authors on reasonable request.
