# Supplementary material for: Trends in patient tobacco use behaviors as reported by tobacco treatment providers: Findings from a repeated crosssectional survey
Source: Tob Prev Cessat. 2025 Oct 20;11:10.18332/tpc/210929. doi: 10.18332/tpc/210929 (PMC12550529; doi:10.18332/tpc/210929)
Supplement: Supplementary file 1 [file TPC-11-47-s1.pdf]

Supplemental Figure 1. Qualtrics questions assessing providers reported patient tobacco use behaviors in the past 6 months.

In the past 6 months, have any patients **mentioned** any of the following tobacco/nicotine products? Do **not** include FDA approved pharmacotherapy (NRT, bupropion, varenicline)

	Yes	No
Heated tobacco products (like IQOS)	○	○
Nicotine gummies/candy	○	○
Cigars, cigarillos, or filtered cigars	○	○
Tobacco-free nicotine lozenges (i.e., not FDA-approved pharmacotherapy)	○	○
Nicotine toothpicks	○	○
Smokeless tobacco [snuff, chew, dip]	○	○
Cigarettes	○	○
Snus	○	○
Non-FDA-approved nicotine gum	○	○
Electronic cigarettes [e-cigarettes] or vaping	○	○
Tobacco-free nicotine pouches (i.e., not FDA-approved pharmacotherapy)	○	○
Hookah	○	○
Other nicotine or tobacco product, please specify	○	○

In the past 6 months, have any patients **sought treatment to stop using** any of the following products?

	Yes	No
Snus	◯	◯
Tobacco free nicotine lozenges (i.e., not FDA-approved pharmacotherapy)	◯	◯
Nicotine gummies/candy	◯	◯
Smokeless tobacco [snuff, chew, dip]	◯	◯
Electronic cigarettes [e-cigarettes] or vaping	◯	◯
Nicotine toothpicks	◯	◯
Non-FDA-approved nicotine gum	◯	◯
Heated tobacco product (like IQOS)	◯	◯
Hookah	◯	◯
Cigars, cigarillos, filtered cigars	◯	◯
Cigarettes	◯	◯
Tobacco-free nicotine pouches (i.e., not FDA-approved pharmacotherapy)	◯	◯
Other nicotine or tobacco product, please specify ________	◯	◯

In the past 6 months, have any of your patients **used one of these to stop using another product (e.g., for smoking cessation)**?

	Yes	No
Electronic cigarettes [e-cigarettes] or vaping	◯	◯
Tobacco free nicotine lozenges (i.e., not FDA-approved pharmacotherapy)	◯	◯
Tobacco-free nicotine pouches (i.e., not FDA-approved pharmacotherapy)	◯	◯
Heated tobacco products (like IQOS)	◯	◯
Cigars, cigarillos, filtered cigars	◯	◯
Nicotine Gummies/Candy	◯	◯
Snus	◯	◯
Cigarettes	◯	◯
Hookah	◯	◯
Nicotine Toothpicks	◯	◯
Smokeless tobacco [snuff, chew, dip]	◯	◯
Non-FDA-approved nicotine gum	◯	◯
Other nicotine or tobacco product, please specify ________	◯	◯

© 2025 Mercincavage M. et al.
